# The Farmer Field School: a method for enhancing the role of rural communities in malaria control ?

**DOI:** 10.1186/1475-2875-5-3

**Published:** 2006-01-19

**Authors:** Henk van den Berg, Bart GJ Knols

**Affiliations:** 1 Laboratory of Entomology, Wageningen University and Research Centre, P.O. Box 8031, 6700 EH, Wageningen, The Netherlands; 2 International Atomic Energy Agency (IAEA), Agency's Laboratories Seibersdorf, Seibersdorf A-2444, Austria

## Abstract

Malaria has strong linkages with agriculture, and farmers in malarious regions have a central position in creating or controlling the conditions that favour disease transmission. An interdisciplinary and integrated approach is needed to involve farmers and more than one sector in control efforts. It is suggested that malaria control can benefit from a complementary intervention in rural development, the Farmer Field School (FFS) on Integrated Pest Management (IPM). This is a form of education that uses experiential learning methods to build farmers' expertise, and has proven farm-level and empowerment effects. The benefits of incorporating malaria control into the IPM curriculum are discussed. An example of a combined health-agriculture curriculum, labeled Integrated Pest and Vector Management (IPVM), developed in Sri Lanka is presented. Institutional ownership and support for IPVM could potentially be spread over several public sectors requiring a process for institutional learning and reform.

## Introduction

Malaria has major and multifaceted linkages with agriculture, both in a rural and peri-urban context [1,2]. Agricultural environments provide conditions well suited for anopheline breeding, with clear, temporary water bodies coinciding with the time of crop cultivation, and human and animal hosts at flying distance. Clearing of land for agriculture opens up breeding habitats for heliophilic vectors, whereas "informal" smallholder farming systems located near natural water sources, such as streams and rivers, open up vector breeding opportunities. Moreover, mounting evidence indicates that the widespread agricultural use of broad-spectrum insecticides contributes to insecticide-resistance in mosquito vectors [3,4]. This is expected to reduce the efficacy of indoor residual spraying and insecticide-treated bednets, although the efficacy of the latter intervention has, so far, only been marginally unaffected even in areas with high frequency of the *kdr *gene in the vector population [5]. In addition, agriculture generates income and, thus, influences living conditions, which can affect the transmission and severity of disease. Malaria, in turn, impedes human workforce output and agricultural production, especially at times that agricultural activities peak (i.e. the time of irrigation or after rains). In the African context, this effect is exemplified by the dual role of women in agricultural activities and as family caregiver.

Central in all these crucial linkages is the position of farmers – who create the agricultural environments, implement the cultivation practices, decide on agrochemical inputs, and who attain, as a result of these actions, a certain living standard. Addressing the malaria-agriculture interface requires, therefore, a broad interdisciplinary and integrated approach that involves local communities and more than one public sector. The importance of active participation and empowerment of rural communities to the effectiveness of malaria control interventions and to the sustainability of their outcomes has been repeatedly stressed [6]. Some progress has been made by making health services better embedded within community structures [7,8] or marketing systems [9], but these developments do not necessarily result in local initiative or the empowerment of communities.

In the area of environmental management, which is receiving renewed interest, past project attempts have shown that it can be difficult to motivate local people into action [10], possibly, as has been suggested, due to the absence of economic incentive mechanisms [6]. There have been examples of successful initiatives [[11-13]], but their sustainability remains an issue. Evidently, experience with participation and empowerment of rural people is most established within the agricultural sector. A variety of interpretations of participation exist, ranging from passive participation (where people are told what is to happen) and functional participation (as a means to achieve external goals), to self-mobilization (where the people take initiative), with the latter most likely to lead to positive change [14]. In this commentary, the potential value of a popular participatory approach for malaria control, called the Farmer Field School, is discussed, while drawing on experience from a project in Sri Lanka.

### Farmer Field School

The Farmer Field School (FFS) has one of the most impressive track records in participatory community approaches [15], with 2–3 million farmers graduated on the agricultural subject of Integrated Pest Management (IPM) during the past 15 years, mainly in Asia, but more recently also in Africa, the Middle East and Latin America [16]. A review of 25 impact studies indicated a range of positive outcomes of IPM Farmer Field Schools including drastic reductions in agro-pesticide use, economic benefits and empowerment effects [17]. The FFS approach evolved from the need to strengthen the ecological basis of Integrated Pest Management (IPM) to deal with the variability and complexity of agro-ecosystems whilst reducing reliance on pesticides. The ecology of opportunist insects (which include mosquitoes) is highly localized and dynamic, with populations fluctuating manyfold both spatially and temporally. Accordingly, most tropical smallholder agro-ecosystems require management decisions that are tailored to local and contemporary conditions. This implies the need to decentralize expertise to the field level by educating local people to analyse field situations and to make appropriate management decisions.

**Figure 1:**
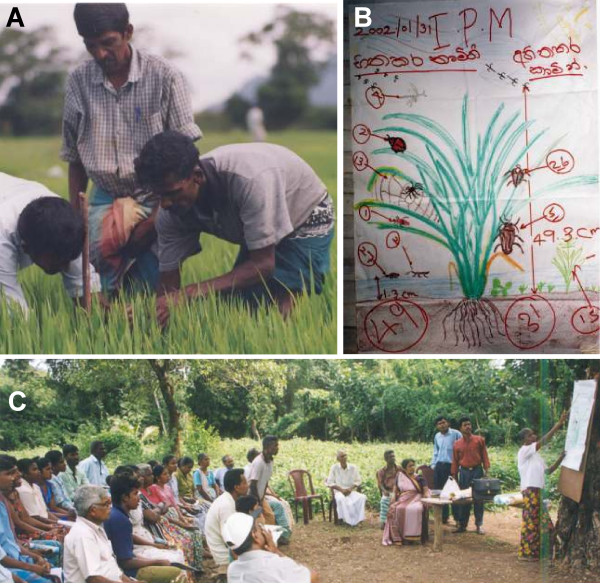
The FFS activity of agro-ecosystem analysis depicted, with the stages of (a) regular field observation, (b) drawing and analysis, and (c) presentation and group discussion. Photographs by H. van den Berg, Sri Lanka, 2002.

The Farmer Field School is a form of education which uses experiential learning methods to build farmers' expertise [18]. In sessions at weekly intervals during a crop cycle, a group of 15–30 neighbouring farmers meet in an open-air situation to take observations of the agro-ecosystem (Figure 1). Several sub-groups of farmers sample the populations and characteristics of harmful and beneficial organisms, plants, soil and environmental conditions. These observations are analysed and presented on newsprint for group discussion, which provides an opportunity for speculation (for example, "what if, instead of spraying, we drain the water to control planthoppers in rice"), leading to decision-making on experimental action to be evaluated in the following week. These weekly completed learning cycles result in strengthened skills and increased confidence of farmers. Several additional observations or experiments are conducted during the field school to study life cycles, insect behaviour, and plant damage. Group dynamics and communication exercises are conducted to strengthen group cohesion, maintain motivation and help participants to develop organizational skills. Group building is important in approaches such as IPM and disease vector management, which benefit from coordinated management by many farmers over a large area. Post FFS support has been given in a number of countries to facilitate the emergence of local project initiative [18].

### Multi-pronged strategy

It is noteworthy that the educational investment in agriculture can indirectly benefit health. Specifically, IPM Farmer Field School programmes located in malarious areas will inadvertently influence malaria epidemiology if income and living standards are raised (by improving people's access to health care) and if agro-pesticide use is reduced (by lowering the risk of insecticide resistance in malaria vectors). IPM programmes may be complementary to malaria control efforts. A coordinated inter-sectoral planning and implementation of activities could potentially make better use of scarce resources and synergise effects. It is proposed that integration could be taken one step further. A logical adaptation of the IPM Farmer Field School is to make the ecology and control of malaria disease implicit in the IPM curriculum, by purposely involving farmers and other interested actors in the management of malaria in their environment.

**Figure 2:**
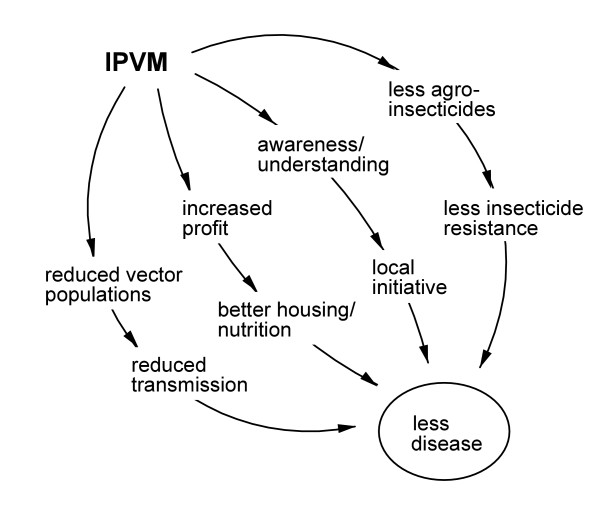
Four possible effects of IPVM Farmer Field Schools on malaria disease: (a) the effect of reduced agro-pesticide use on the risk of insecticide resistance in vector mosquitoes; (b) the effect of increased awareness and understanding about malaria on personal protection measures and treatment-seeking behaviour; (b) the effect of increased profits from agriculture on nutrition and housing conditions; (d) the effect of environmental management on vector breeding and thus on the transmission of disease.

This combined strategy, labeled Integrated Pest and Vector Management (IPVM), can be expected to affect malaria disease in four different ways (Figure 2). First, as mentioned above, a reduced use of agro-pesticides, the most consistent outcome of IPM Farmer Field School programmes, will lower the risk of resistance in the vector. Second, experiential learning leads to practical knowledge about the ecology and epidemiology of malaria, which could influence personal protection measures or treatment-seeking behaviour. Third, IPM results in increased income from agriculture, due to increased yield and due to savings in pesticide expenditure [17]. An economic outcome may benefit nutrition, housing conditions and access to health services. Fourth, environmental management by farming communities is expected to suppress mosquito breeding and, consequently, to reduce the period and/or intensity of disease transmission. Critical issues in environmental management are the portion of breeding sites covered, the local landscape and possible increased fitness or survival of the vector at reduced population density. Reduction of nuisance mosquitoes (which is in itself a benefit), could affect bednet use unless this matter is taken up in the FFS curriculum.

This broad range of outcomes implies that the IPVM methodology is not necessarily restricted to situations where prospects for environmental management exist. IPVM could be targeted to address rural poverty, awareness or pesticide over-use, even in areas where environmental management is not an option.

### Rice as starting point

Synergistic relationships between rice cultivation and vector management have long been known [19], which makes wetland rice a promising starting point for an integrated strategy. Wetland rice can produce large vector populations, though not necessarily of high vectorial capacity. Interruption of vector breeding in leveled fields is often technically feasible through regular drainage [20] or through appropriate management during the fallow period [21]. Considering the flight radius of anophelines, however, field-level management would need to be implemented over a sufficient area to have an effect on vector populations. This requires active participation of large numbers of farmers. In many irrigation situations, water management is insufficiently controlled by individual farmers, although this is potentially improved through the FFS and its socio-political impacts.

In epidemic-prone areas at the margins of malaria transmission, or in areas with strong seasonal malaria, a reduced period or intensity of transmission is expected to cause a temporary decline in malarial disease, because parasitaemia usually results in disease in non-immune people. In parts of Africa with stable holoendemic malaria, a reduced transmission is expected to reduce the risk of severe disease, particularly in infants and young children before they have acquired a certain level of natural immunity [22]. As far as possible, environmental management of malaria needs to be conducted alongside other locally-appropriate control interventions, under an evidence-based Integrated Vector Management (IVM) approach [6]. Ultimately, the impact of community-based activities on local transmission and morbidity of malaria needs to be assessed. This requires locally clustered field school programmes to achieve an appropriate scale, a control and local measurements of transmission and morbidity.

### Adapted curriculum

The IPVM Farmer Field School curriculum has recently been developed for the wetland rice ecosystem in Sri Lanka. The project, involving one of us (HvdB), built on the experience of an existing IPM project. Experiential learning exercises on the health component have been developed to complement, not replace, the existing curriculum on IPM.

The process of curriculum development was mostly in the hands of selected IPM facilitators with technical support from health resource persons. Learning objectives for the new curriculum were based on surveys, which were conducted by the facilitators to study the perceptions and knowledge of rural people about vector-borne diseases, in particular malaria. The developed learning exercises used simple and easily replicable methods, as well as locally available materials. The health component was addressed mainly in the first part of the season, when most anopheline breeding takes place. Different gender roles were noted by the facilitators, women showing most interest in the health component and men in the agricultural component. Preliminary observations in Sri Lanka suggest that farmers can be motivated and educated to play an active and competent role in vector management. Similar curricula could be included in formal school education in rural areas.

Details of the learning exercises are given in Table 1. Sampling of mosquito developmental stages and aquatic predators was incorporated within the weekly exercise of agro-ecosystem analysis. The exercise was initiated well before planting and continued as long as standing water was available in the field; post-harvest larval breeding had not been identified as an important issue. Farmers learned to recognize the larvae and adults of the three main mosquito genera, *Anopheles*, *Culex *and *Aedes*, using behavioural and taxonomic clues, to study mosquito-breeding habitat, and to monitor adult mosquitoes. Mosquito life cycles and mosquito predators were studied by daily observations inside transparent containers. Land leveling and alternate wet-dry irrigation were implemented in the study plots to manage mosquitoes, and farmers learned to practice source reduction, through filling, draining or manipulating water bodies in their environment. This involved planning, mapping and action by the group of participating farmers. The disease cycle of malaria, a topic unsuitable for practical learning, was addressed through a role-play, whereby farmers were assigned roles of human hosts, vector and parasite stages, and whereby the process of transmission was performed.

**Table 1:** Exercises on mosquito biology, ecology and management which were added to the agricultural topics of the Farmer Field School curriculum.

Exercise	Methods	Purpose
1 Agro-ecosystem analysis	Comprehensive sampling of the crop ecosystem (using soup spoons, visual counts, plant measurements, etc.) and visual presentation and analysis	To monitor the agro-ecosystem and make context-specific decisions on necessary action related to crop production and human health
2 Mosquito breeding habitat	Dipper sampling in, and characterization of, various aquatic habitats of mosquito genera	To study where different mosquito genera breed
3 Adult mosquito sampling	Collecting adult mosquitoes at different times and habitats and identify major genera, i.a. using home-made aspirators	To monitor potential disease vectors and their activity
4 Mosquito lifecycle	Rearing of young larvae in water jar covered with mesh	To understand the relation between maggot, pupae and adult, and the development time
5 Mosquito identification	Observing larval behaviour and adult characteristics	To distinguish *Anopheles*, *Aedes *and *Culex *in the larval and adult stage
6 Predation	Exposing larvae or pupae to a range of arthropods inside jars	To understand the role of predators in controlling mosquito developmental stages
7 Analogy on disease cycle	Role play on the cycle of the parasite through human and mosquito hosts	To understand the role of the vector and the human reservoir
8 Agricultural methods to suppress mosquito breeding	Alternate wet-dry irrigation of study field plots; land levelling at planting	To study how farmer practices influence mosquito breeding and crop development
9 Source reduction	Farmer action to drain or fill water bodies, including in the peri-domestic environment	To practice measures to contain vector breeding
10 Mapping	Drawing map of village environment with water bodies, crops, houses, etc.	To facilitate planning for coordinated action on environmental management

## Conclusion

As managers of their rural environment, farmers have a role to play in malaria control. An approach aiming to empower and organize farmers to take initiative is presented. The IPVM Farmer Field School connects the health component to agricultural productivity, which broadens the institutional basis of malaria control and provides an economic incentive mechanism necessary to motivate people into action. Adaptation of the approach to other agro-ecosystems with associated vector breeding (e.g. irrigated vegetables, cotton, ridge crops) requires further study. The approach has potential to raise living standards of the rural poor above a threshold level at which people have the means to combat malaria. As educational investment, the IPVM Farmer Field School is expected to cause a lasting and profound change in people and in their relationship with the environment. Costs of up-scaling field school activities do not necessarily come from projects or public health budgets, but institutional ownership and support could potentially be shared by sectors of health, agriculture, education, irrigation and environment. Hence, a process for institutional learning and reform needs to be initiated.
